# Method for absolute quantification of short chain fatty acids via reverse phase chromatography mass spectrometry

**DOI:** 10.1371/journal.pone.0267093

**Published:** 2022-04-20

**Authors:** Dominique G. Bihan, Thomas Rydzak, Madeleine Wyss, Keir Pittman, Kathy D. McCoy, Ian A. Lewis

**Affiliations:** 1 Department of Biological Sciences, University of Calgary, Calgary, Alberta, Canada; 2 Department of Physiology and Pharmacology, Snyder Institute for Chronic Diseases, Cumming School of Medicine, University of Calgary, Calgary, AB, Canada; University of Pisa, ITALY

## Abstract

Short chain fatty acids (SCFAs; including acetate, propionate, and butyrate) are an important class of biological molecules that play a major role in modulating host-microbiome interactions. Despite significant research into SCFA-mediated biological mechanisms, absolute quantification of these molecules in their native form by liquid chromatography mass spectrometry is challenging due to their relatively poor chromatographic properties. Herein, we introduce SQUAD, an isotope-based strategy for absolute quantification of SCFAs in complex biological samples. SQUAD uses aniline derivatization in conjunction with isotope dilution and analysis by reverse-phase liquid chromatography mass spectrometry. We show that SQUAD enables absolute quantification of biologically relevant SCFAs in complex biological samples with a lower limit of detection of 40 nM and a lower limit of quantification ranging from 160 nM to 310 nM. We observed an intra- and inter-day precision under 3% (relative standard deviation) and errors in intra- and inter-day accuracy under 10%. To demonstrate this quantification strategy, we analyzed SCFAs in the caecal contents of germ free versus conventionally raised specific pathogen free (SPF) mice. We showed that acetate was the most abundant SCFA in both types of mice and was present at 200-fold higher concentration in the SPF mice. We also illustrated the use of our quantification strategy in *in vitro* microbial cultures from five different species of bacteria grown in Mueller Hinton media. This study illustrates the diverse SCFA production rates across microbial taxa with acetate production serving as one of the key differentiating factors across the species. In summary, we introduce an isotope dilution strategy for absolute quantification of aniline-dativized SCFAs and illustrate the utility of this approach for microbiome research.

## Introduction

Short chain fatty acids (SCFAs) are produced by the fermentation of indigestible dietary fiber by the commensal gut microbiota. Acetate (Ac), propionate (Prop), and butyrate (But) make up the majority of SCFAs in the gut, and are present at various ratios throughout the mammalian gastrointestinal tract [1–3]. SCFAs are known to directly influence both the epigenome and the immune system, and play an important role in host energy balance [4–6]. They have thus become an important topic in microbiome-based research. Accurately quantifying the abundance of SCFAs is therefore an important objective for a wide transect of researchers investigating the interaction between gut microbiota and the host.

The analysis of SCFAs has been accomplished using a number of analytical techniques. Traditionally, gas chromatography (GC) coupled to a number of detection platforms has served as the primary analytical strategy [7–10]. Though effective, these GC-based analytical approaches are not transferable to liquid chromatography mass spectrometry (LC-MS), which has become the preferred platform used by most metabolomics-oriented analytical facilities. Unfortunately, SCFAs are challenging for LC-MS due to their poor chromatographic and ionization properties on conventional LC-MS instrumentation [11]. These challenges have led several groups to develop alternative approaches for profiling SCFAs and other related molecules [12–15].

One of the main analytical strategies for making SCFAs compatible with LC-MS has been to chemically derivatize samples to improve the chromatographic separation and MS properties of SCFAs (e.g., parent mass and ionization efficacy). Two successful strategies have included derivatization by 3-nitrophenylhydrazines [13] and aniline [16]. Both methods produce high yields and enable SCFA analyses to be conducted on conventional C18 chromatography. These methods are also convenient because they are compatible with LC solvents and are conducted under relatively mild reaction conditions.

Both of the established SCFA derivatization methods use a strategy wherein calibration standards are prepared independently from the test samples. In one case [16] these standards are derivatized with an isotope-labeled reagent and are added to a matrix-matched sample to serve as an external standard reference. In the other case [13] the isotope-labeled derivatized internal standards are added after samples have been extracted and derivatized. These derivatization strategies allow readily-available ^12^C commercial molecules to be used as standards and correct for MS-related variables [17], respectively. Although this approach provides the greatest possible scope for metabolomics applications [13,16,18], derivatizing standards independently from the test sample introduces several potentially significant sources of quantitative error. Most importantly, any differences in derivatization efficiency between standards and test samples will directly affect quantification. Moreover, internal standards added after the sample preparation process cannot correct for errors associated with extraction efficiency and sample handling. Although these sources of error can be controlled by carefully monitoring and optimizing the sample preparation process, these practices are challenging for routine biological applications.

The primary objective of this study was to develop a practical SCFA quantification method that corrects for the greatest scope of errors while minimizing experimental complexity. Importantly, most biological studies related to SCFAs have focused on just three molecules (acetate, propionate, and butyrate) [19], all of which are commercially available as ^13^C-labeled compounds. With this practical consideration in mind, we propose an alternative stable isotope dilution (SID) workflow [20,21] for absolute quantification of SCFAs in biological samples. The primary advantage of our workflow over the established methods is that isotope-labeled standards are added prior to any sample handling. This approach supports robust LC-MS quantification of SCFAs irrespective of differences in derivatization efficiency and sample handling. Herein we validate this method, measure limits of detection, investigate potential sources of experimental error, provide guidelines for conducting biological studies using this approach, and apply the method to two typical biological studies in which SCFA analysis is important.

## Materials and methods

### Materials and reagents

Unless otherwise specified, all reagents were purchased from Sigma-Aldrich (St. Louis, MO, USA). Water (H_2_O, # W6), methanol (MeOH, # A454) and acetonitrile (ACN, # A996) Optima^TM^ grade HPLC solvents were purchased from Fisher Scientific, Inc (Hampton, NH, USA). Formic acid (# 33015, ACS reagent grade). ^12^C- and ^13^C-SCFA standards used in this study were as follows: acetic acid, glacial (# AX0073, EDM Millipore, Burlington, MA, USA), propionic acid (# 94425), butyric acid (# 19215), isobutyric acid (# 46935-U), valeric acid (# 75054), isovaleric acid (# 78651), 2-methylbutyric acid (# 49659), acetic acid (1,2-13C_2_, 99%) (# CLM-113, Cambridge Isotope Laboratories, Inc (Andover, MA, USA)), propionic acid-13C_3_ (99 atom % ^13^C) (# 589586), butyric acid-1,2-13C_2_ (99 atom % ^13^C) (# 491993). Deuterated internal standards isobutyric-d7 acid (# I789183), valericd9 acid (# V091417) and isovaleric-d9 acid (# I917572) were purchased from Toronto Research Chemicals (Toronto ON, Canada). Aniline (# 242284) and N-(3-Dimethylaminopropyl)-N′-ethylcarbodiimide hydrochloride (EDC) (# E6383) were obtained from Sigma-Aldrich.

### Derivatization of ^12^C/^13^C SCFA standard mix solution

Derivatization was performed by adding 5 μL of a 2.4 M aniline solution (in ACN), followed by 5 μL of a 1.2 M EDC solution (in H_2_O) to 100 μL of a ^12^C/^13^C SCFA standard mix solution containing 6 SCFA standards (^12^C-acetate,-propionate and -butyrate, and their corresponding ^13^C-counterparts, at a final concentration of 1 mM each) in H_2_O/ACN (50:50, v/v) cooled to 0°C. The reaction mixture was kept on ice for 2 hours, with regular mixing, following which an aliquot was diluted in H_2_O/MeOH (50:50, v/v) and submitted to LC-MS/MS analysis.

### Preparation of calibration curves

A standard mix solution containing all 6 SCFA standards (^12^C-acetate, -propionate and -butyrate, and their corresponding ^13^C-counterparts) at a final concentration of 1 mM each was prepared in H_2_O/ACN (50:50, v/v). A 100 μL aliquot was derivatized as described above and was then diluted (1:10 dilution) with H_2_O/MeOH (50:50, v/v) to prepare 15 solutions with a concentration range of 100 μM to 20 nM.

### Preparation of quantification test mixtures

Samples were prepared from 0.2 M ^12^C-SCFA (0.5 mL) and 0.01 M ^13^C-SCFA (2 mL) stock solutions, both in H_2_O/ACN (50:50, v/v). Solutions were diluted and mixed to give 29 samples with a defined [^12^C-SCFA]:[^13^C-SCFA] ratio ranging from 0.001 to 1000 (see S1 Table). The derivatization reaction was carried out as described above, then samples were diluted 1:100 (v/v) in H_2_O/MeOH (50:50, v/v) and submitted to LC-MS/MS analysis.

### Animal procedures

SPF C57BL/6(J) mice were bred and maintained in the specific pathogen-free facility at the University of Calgary Animal Resource Centre. Germ free C57BL/6 mice were bred and maintained in flexible film isolators at the International Microbiome Centre (IMC) at the University of Calgary. Germ-free status was routinely monitored by culture-dependent and independent methods and all mice were independently confirmed to be pathogen-free. 10–12 week old mice were used in this study. Animals were humanely euthanized and caecal contents were removed via a longitudinal incision of the caecal wall. Contents were immediately snap-frozen in liquid nitrogen and stored at -80°C until processed. Animals were euthanized with an overdose of isoflurane followed by cervical dislocation. All animal experiments were approved by the University of Calgary Animal Care Committee (protocols AC16-0235, AC17-0011, and AC17-0090) and were performed in accordance with the guidelines established by the Canadian Council for Animal Care.

### Analysis of SCFAs in caecal samples

Caecal samples were removed from -80°C storage and placed on ice. Ice-cold extraction solvent (H_2_O/ACN (50:50, v/v)) containing ^13^C-SCFA internal standards (IS) was added to each sample, as detailed in S2 Table. Samples were vortex-mixed for 3 min then centrifuged at 18 000 × *g* for 10 min at 4°C. Supernatants were collected, submitted to another centrifugation step and then derivatized as follows: to 50 μL of supernatant cooled at 0°C, was added 2.5 μL of a 2.4 M aniline solution (in ACN), followed by 2.5 μL of a 1.2 M EDC (in H_2_O). The reaction mixture was kept on ice for 2 hours, with regular mixing. After 2 hours, an aliquot was diluted 1:200 (v/v) for SPF samples in H_2_O/MeOH (50:50, v/v) and submitted for LC-MS/MS analysis. GF samples were analyzed undiluted.

### Analysis of SCFAs in microbial cultures

All bacterial strains were clinical isolates acquired from APEX Isolate Biorepository at the University of Calgary. Bacterial samples were grown in 96-well culture plates (Corning, New York, NY, USA) containing Mueller Hinton medium. Exponential phase bacterial cultures were used to seed medium to a starting OD_600_ ~ 0.07. Cultures were incubated in a humidified incubator (Heracell VIOS 250i Tri-Gas Incubator, Thermo Scientific, Waltham, MA, USA) under a 5% CO_2_ and 21% O_2_ atmosphere for 4 hours. Growth was measured at OD_600_ (Multiskan GO, Thermo Fisher Scientific, Waltham, MA, USA). After incubation, samples were transferred to a 96-well PCR plate (VWR), and centrifuged for 10 min at 4000 × *g* at 4°C to remove cells. Supernatant was removed, mixed 1:1 with 100% LC-MS grade MeOH, and either frozen at -80°C for further processing, or centrifuged again for 10 min at 4000 × *g* at 4°C to remove any protein precipitate. 20 μL of each supernatant were then dispensed into a 96-well plate. 5 μL of the IS solution in H_2_O/MeOH (50:50, v/v), with final concentrations of 2 mM for ^13^C-acetate, 5 μM for ^13^C-propionate, and 25 μM for ^13^C-butyrate, was added to each well, followed by 1.25 μL of aniline solution (2.4 M, in MeOH) and 1.25 μL of EDC solution (1.2 M, in H_2_O). Samples were kept at 0°C for 2 hours with regular shaking, then diluted 1:8 (v/v) in H_2_O/MeOH (50:50, v/v) and submitted for LC-MS/MS analysis. Reported SCFA concentrations have been corrected for sample dilutions before derivatization.

### Evaluation of derivatization conditions

#### Effect of the EDC molar equivalent on observed ^12^C:^13^C ratio

Five different molar equivalents (0.1, 0.5, 1, 5 and 10) of EDC were each tested on six different ^12^C/^13^C SCFA standard mix solutions with a ^12^C:^13^C concentration ratio of 0.5, 0.8, 1, 1.25, 2 and 5 prepared as described in S1 Table. The number of moles of EDC (see S4 Table for further details) was calculated based on the total number of moles of SCFAs present in each standard mix (ranging from 0.36 to 0.6 μmole). Five concentrations of EDC and aniline solutions were prepared (0.012, 0.06, 0.12, 0.6 and 1.2 M for EDC, and 0.024, 0.012, 0.24 1.2 and 2.4 M for aniline). The added volume of reagents was adjusted (ranging from 3 to 5 μL) to add the exact number of mole equivalents within the six standard mixtures. The derivatization reaction was carried out as described above. Prior to LC-MS/MS analysis, aliquots of the reaction mixtures were diluted 1:100 with H_2_O/MeOH (50:50, v/v). Reaction mixtures corresponding to a low EDC molar equivalent (0.1 and 0.5), and therefore containing a low concentration of derivatized analytes, were diluted 1:10 with H_2_O/MeOH (50:50, v/v).

#### Effect of the reaction time on observed ^12^C:^13^C ratio

Six different ^12^C/^13^C SCFA standard mix solutions with a ^12^C:^13^C concentration ratio of 0.5, 0.8, 1, 1.25, 2 and 5 were prepared in H_2_O/ACN (50:50, v/v) (see S1 Table) and submitted to a derivatization step as described above. Aliquots (10 μL) were sampled over time, diluted 100 times in H_2_O/MeOH (50:50, v/v) and kept at 0°C until LC-MS/MS analysis on the same day.

#### Effect of the reaction solvent on observed ^12^C:^13^C ratio

Four different ^12^C/^13^C SCFA standard mix solutions, containing ^12^C-acetate, ^12^C -propionate and their corresponding ^13^C-counterparts, were prepared with a ^12^C:^13^C concentration ratio of 0.5, 1, 2 and 5. The final concentration of each analyte in each (^12^C-SCFA:^13^C-SCFA) solution was (500 μM:1 mM), (50 μM:50 μM), (500 μM:250 μM) and (250 μM:50 μM) respectively. Each (^12^C-SCFA:^13^C-SCFA) solution was prepared in five different reaction/organic solvents, namely H_2_O/ACN (50:50, v/v), H_2_O/ACN (20:80, v/v), H_2_O/MeOH (50:50, v/v), H_2_O/MeOH (20:80, v/v) and H_2_O/MeOH/ACN (20:40:40, v/v). The derivatization step was carried out as described above. Prior to LC-MS/MS analysis, samples were diluted, with H_2_O/MeOH (50:50, v/v), as follows: 1:100 for the (500 μM:1 mM) solution, 1:50 for the (500 μM:250 μM) solution and 1:10 for the (50 μM:50 μM) and (250 μM:50 μM) solutions.

### LC-MS/MS sample analysis

LC-MS/MS analysis was performed on a Vanquish^TM^ ultra high performance liquid chromatography (UHPLC) system coupled to a TSQ Quantum^TM^ Access MAX triple quadrupole mass spectrometer (Thermo Fisher Scientific) equipped with an electrospray ionization (HESI-II) probe. The UHPLC-MS platform was controlled by an Xcalibur^TM^ data system (Thermo Fisher Scientific). Chromatographic separation was achieved on a Hypersil GOLD ^TM^ C18 column (200 X 2.1 mm, 1.9 μm, Thermo Fisher Scientific) using a binary solvent system composed of LC-MS grade H_2_O with 0.1% (%v/v) formic acid (Solvent A) and LC-MS grade MeOH with 0.1% (%v/v) formic acid (solvent B). The following 21 min gradient was used: 0–1 min, 10% B; 1–1.1 min, 10–40% B; 1.1–11 min, 40–98% B; 11–16 min, 98% B; 16–16.5 min, 98–10% B, 16.5–21 min, 10% B. LC eluent was diverted to waste for the first 5 min of the run. The flow rate was 200 μL min^-1^ and the sample injection volume 2 μL. The auto sampler was kept at 4°C and the column at 30°C.

**Fig 1:**
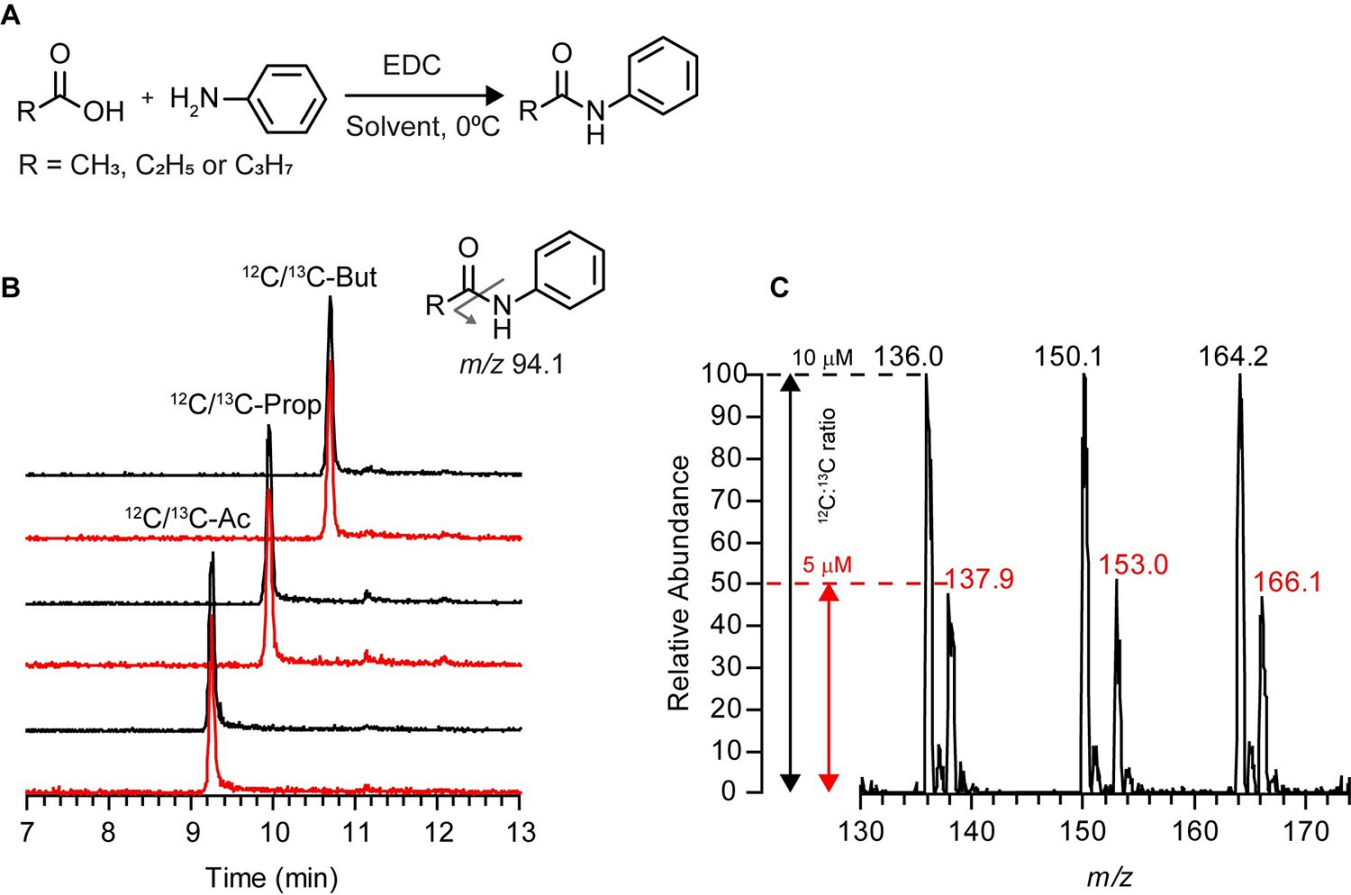
Analytical characterization of derivatized SCFA standards. (A) Reaction scheme for the aniline derivatization of SCFAs. (B) Overlaid extracted ion chromatograms (EIC) corresponding to a ^12^C-and ^13^C-derivatized SCFA mix standard solution detected in selected reaction monitoring mode. Red HPLC traces correspond to the ^13^C-labeled analytes. (Inset) A single reporter MS/MS fragment was used for analyzing all SCFA: *m/z* [M+H]^+^ → 94.1. (C) Relative MS signal intensities of a derivatized mixed solution of ^12^C- and ^13^C- SCFA analytes at a concentration of 10 μM and 5 μM, respectively. Data were acquired in full scan mode. EDC, N-(3-Dimethylaminopropyl)-N′-ethylcarbodiimide hydrochloride; CE, collision energy in eV.

MS/MS data were acquired in positive electrospray ionization mode with the mass spectrometer operating in selected reaction monitoring (SRM) mode. Fragmentation parameters were optimized using the EZ Tune program with direct infusion of the derivatized analytical grade standards (50 μM each in H_2_O/MeOH (50:50, v/v)). The derivatized ^12^C- and ^13^C-standards have similar breakdown curves, with the most abundant fragment ion detected (*m/z* 94.1; protonated aniline (C6H8N)) resulting from the cleavage of the amide bond, as illustrated in Fig 1B, at a collision energy (CE) of 14-17eV. The second most abundant fragment ion detected, with a relative intensity of 20–40%, was *m/z* 77.0 (28-33eV CE) characteristic of the benzene group (C6H5). Similar results were obtained for the derivatized deuterated standards except that the aniline fragment ion was *m/z* 95.1 at CE 18eV. Subsequently, the following transitions, corresponding to the derivatized ^12^C-SCFAs and ^13^C-internal standards, were monitored, with a scan time of 0.05 sec and a fixed collision energy of 14eV: [M+H]^+^ *m/z* 136.1 (Ac), 138.1 (^13^C-Ac), 150.1 (Prop), 153.1 (^13^C-Prop), 164.1 (But and Isobut), 166.1 (^13^C-But), 178.1 (Val, Isoval and 2-Mebut) → *m/z* 94.1; and for the deuterated internal standards: [M+H]^+^ *m/z* 171.2 (Isobut-d7), 187.2 (Val-d9 and Isoval-d9) → *m/z* 95.1 (18eV for the collision energy). Electrospray ionization source conditions were as follows: spray voltage of 3000 V, vaporizer temperature of 325°C, sheath gas of 35 psi, auxiliary gas flow of 10 (arbitrary units) and sweep gas flow of 2 (arbitrary units), capillary temperature of 275°C.

Data analyses, on the converted mzXML files, were conducted in MAVEN [22,23]. In short, for each SCFA, the determination of the ^12^C-SCFA concentration was based on the isotopic ratio (signal intensity, area under the curve) and the respective known stable isotope-labeled internal standard concentration.

The determination of the lower limit of detection (LLOD) and lower limit of quantification (LLOQ) was based on the analysis of the peak’s height for the analyte signal (S) and the baseline noise (N) of chromatographic peaks corresponding to samples prepared as detailed in “preparation of calibration curves” section, except that only ^12^C-Acetate, -propionate and -butyrate were used. The final concentration of each solution tested was as follows: 0.02, 0.04, 0.08, 0.16 and 0.31 μM. Each sample was injected in triplicates. LLOD and LLOQ were defined as the concentrations where the signal-to-noise (S/N) was greater than or equal to 3 and greater than or equal to 10 respectively (S1C Fig).

For the evaluation of the precision and accuracy of the analytical method, two different ^12^C/^13^C SCFA standard mix solutions, containing ^12^C-acetate, ^12^C -propionate and ^12^C -butyrate, and their corresponding ^13^C-counterparts were prepared, in H_2_O/ACN (50:50, v/v), with a ^12^C:^13^C concentration ratio of 0.5 and 1. The final concentration of each analyte in each (^12^C-SCFA:^13^C-SCFA) solution was (1 mM:2 mM), and (1 mM:1 mM), respectively. The derivatization step was carried out as described above. Prior to LC-MS/MS analysis, samples were diluted 1:100, with H_2_O/MeOH (50:50, v/v), to give two solutions of (10 μM:20 μM), and (10 μM:10 μM), respectively. The precision of the analytical method was estimated by calculating the relative standard deviation (RSD, expressed in percentage), whereas the determination of accuracy was evaluated by calculating the percent error (%Error), both based on the analysis of replicate injections of the sample (20 replicate injections for intra-day variation determination). For inter-day variation determination, data were collected over three consecutive days.

## Results and discussion

### Method for quantifying SCFAs by LC-MS/MS

A defined mixture of ^12^C-acetate, propionate, and butyrate standards (1 mM each, final concentration) and their corresponding^13^C-labeled counterparts (0.5 mM each, final concentration) was subjected to derivatization with aniline, as depicted in the reaction scheme (Fig 1A) and submitted to LC-MS/MS analysis. As expected, the ^13^C-labeled and unlabeled derivatized SCFA analytes co-eluted (Fig 1B), were easily distinguishable by *m/z* (Fig 1C), and had peak intensities proportional to the relative concentrations of the ^12^C:^13^C isotope ratios.

To determine the linear range of detection for both ^12^C- and ^13^C-labeled aniline-derivatized SCFAs using our LC-MS/MS method, calibration curves were generated using an equimolar standard mix solution. Linear relationships were apparent for ^12^C- and ^13^C-labeled acetate, propionate, and butyrate derivatized standards (S1 Fig; r^2^ > 0.995 for all six standards). The slopes of the regression lines for all isotopologues were similar (4.24E+06 / 4.38E+06 for ^12^C/^13^C-Ac, 7.12E+06 / 8.16E+06 for ^12^C/^13^C-Prop and 1.01E+07 / 1.06E+07 for ^12^C/^13^C-But, see S1 Fig). The lower limit of detection (LLOD) and the lower limit of quantification (LLOQ) for all three selected SCFAs were determined with standards derivatized in H_2_O/ACN (50:50, v/v). The LLOD was 40 nM for Ac, Prop and But. The LLOQ for Ac was 0.31 μM, and 0.16 μM for Prop and But (Table 1, S1C Fig). As the quantification of SCFAs in the developed method is based on the accurate determination of the 12C:13C ratio of analytes, the intra- and inter-day precision and accuracy were evaluated using samples, derivatized in H_2_O/ACN (50:50, v/v), with two different known [^12^C-SCFA:^13^C-SCFA] ratios, namely 0.5 and 1. The concentrations of analytes in the corresponding (^12^C-SCFA:^13^C-SCFA) solutions were (10 μM: 20 μM) and (10 μM:10 μM), respectively. The intra-day method precision, expressed as percent relative standard derivatization, was between 2–3%, whereas the inter-day precision (assessed over 3 days) was less than 1%. The accuracy of the method, expressed as percent error, ranged from 0.7 to 10% and 0.2 to 10% for intra- and inter-day accuracy, respectively (Table 2).

**Table 1:** LLOD and LLOQ for the selected SCFAs.

Analyte	LLOD (μM)	LLOQ (μM)
Acetic acid	0.040	0.31
Propionic acid	0.040	0.16
Butyric acid	0.040	0.16

Abbreviations: LLOD, lower limit of detection. LLOQ, lower limit of quantification.

**Table 2:** Intra-and inter-day precision (%RSD) and accuracy (%Error) of the LC-MS/MS method.

		Intra-day (n = 20)			Inter-day (n = 60)		
	Known	Observed ^12^C:^13^C SCFA ratio			Observed ^12^C:^13^C SCFA ratio		
Analyte	[^12^C-SCFA]: [^13^C-SCFA]	Mean ± SD	%RSD	%Error	Mean ± SD	%RSD	%Error
Acetate	1	1.07 ± 0.03	2.96	6.57	1.06 ± 0.01	0.89	5.52
	0.5	0.55 ± 0.01	2.62	10.35	0.548± 0.004	0.683	9.558
Propionate	1	1.04 ± 0.03	3.10	4.07	1.05 ± 0.01	0.52	4.69
	0.5	0.53 ± 0.02	3.07	6.20	0.537 ± 0.005	0.904	7.322
Butyrate	1	0.99 ± 0.02	2.13	0.90	1.00 ± 0.01	0.70	0.18
	0.5	0.50 ± 0.01	2.11	0.69	0.499 ± 0.002	0.427	0.205

Abbreviations: SD, standard deviation. % RSD, percent relative standard deviation. %Error, percent error.

### Sources of error affecting quantification

**Fig 2:**
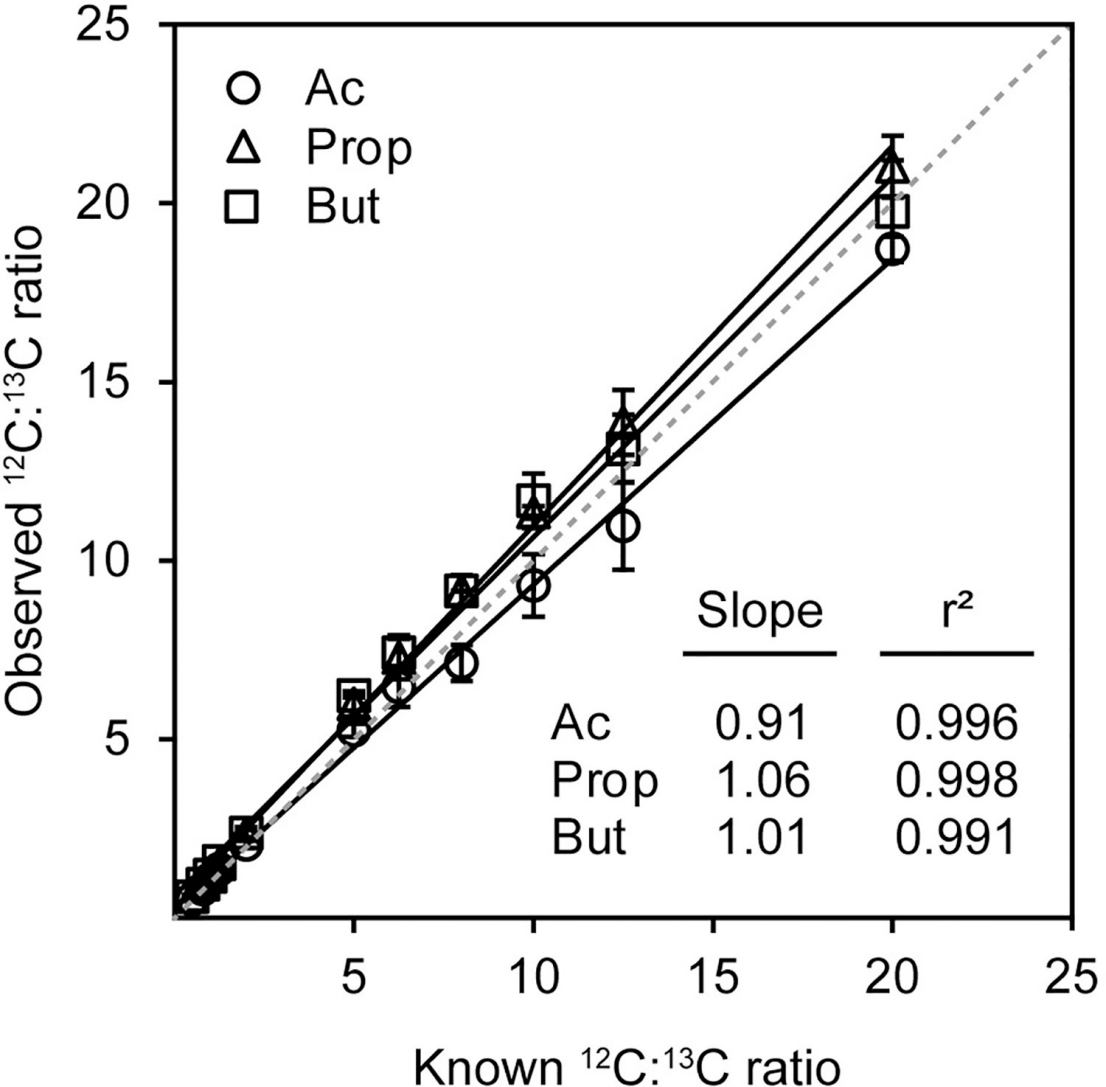
Correlation between known and observed ^12^C:^13^C ratios. Plot of linear regression for known ^12^C:^13^C ratios versus observed ^12^C:^13^C ratios for 11 standard mix solutions with ^12^C- and ^13^C- SCFAs concentration ratios ranging from 0.5 to 20. Slopes for fitted regression lines and r^2^ corresponding to plots are shown. Dashed line represents a slope of 1. Error bars represent standard deviation, *n* = 3 technical replicates.

Derivatization-based quantification strategies coupled to external calibration are frequently subject to quantitative errors resulting from incomplete reactions, differences in reaction efficiencies between samples, and other chemistry-related variables. We used a stable isotope-based dilution strategy—referred to herein as SQUAD (**S**CFA **Q**uantification **U**sing **A**niline **D**erivatization)—to correct for these sources of error. To assess the efficacy of SQUAD, we prepared 11 mixed solutions with ^12^C-and ^13^C-standard concentration ratios ranging from 0.5 to 20. Following derivatization, these samples were analyzed by LC-MS/MS. As expected, isotope ratios observed by LC-MS/MS (signal intensity, area under the curve) matched the known mixing ratios (concentration) for each compound (Fig 2, S3 Table). The reported slopes indicate a percent error ≤ 10% between the theoretical and measured values associated with the range of ^12^C:^13^C concentration ratios tested.

Moreover, a panel of experimental variables that can affect the efficiency of the derivatization (reagent molar equivalents, reaction time and reaction solvents) was evaluated and found to have no significant difference on the quantitative performance of our SQUAD strategy (Table 3, S2–S4 Figs). Those data demonstrate that even in sub-optimal derivatization conditions the ratio of ^12^C- and corresponding ^13^C-analytes is still accurately determined.

**Table 3:** Summary of tested experimental parameters.

Variable	Range	Slope (r^2^)	Figures
EDC^a^ molar equivalent.	0.1–10	0.86–0.95^b^ (> 0.99)	S2
Reaction time	10–180 min	0.89–1.14^b^ (> 0.99)	S3
Extraction solvent	CH_3_CN, MeOH	0.90–1.03^c^ (> 0.99)	S4

^a^ molar equivalent compared to total SCFAs number of moles.

^b^ range of slopes for Ac, Prop and But (averaged data).

^c^ range of slopes for Ac and Prop (averaged data).

**Fig 3:**
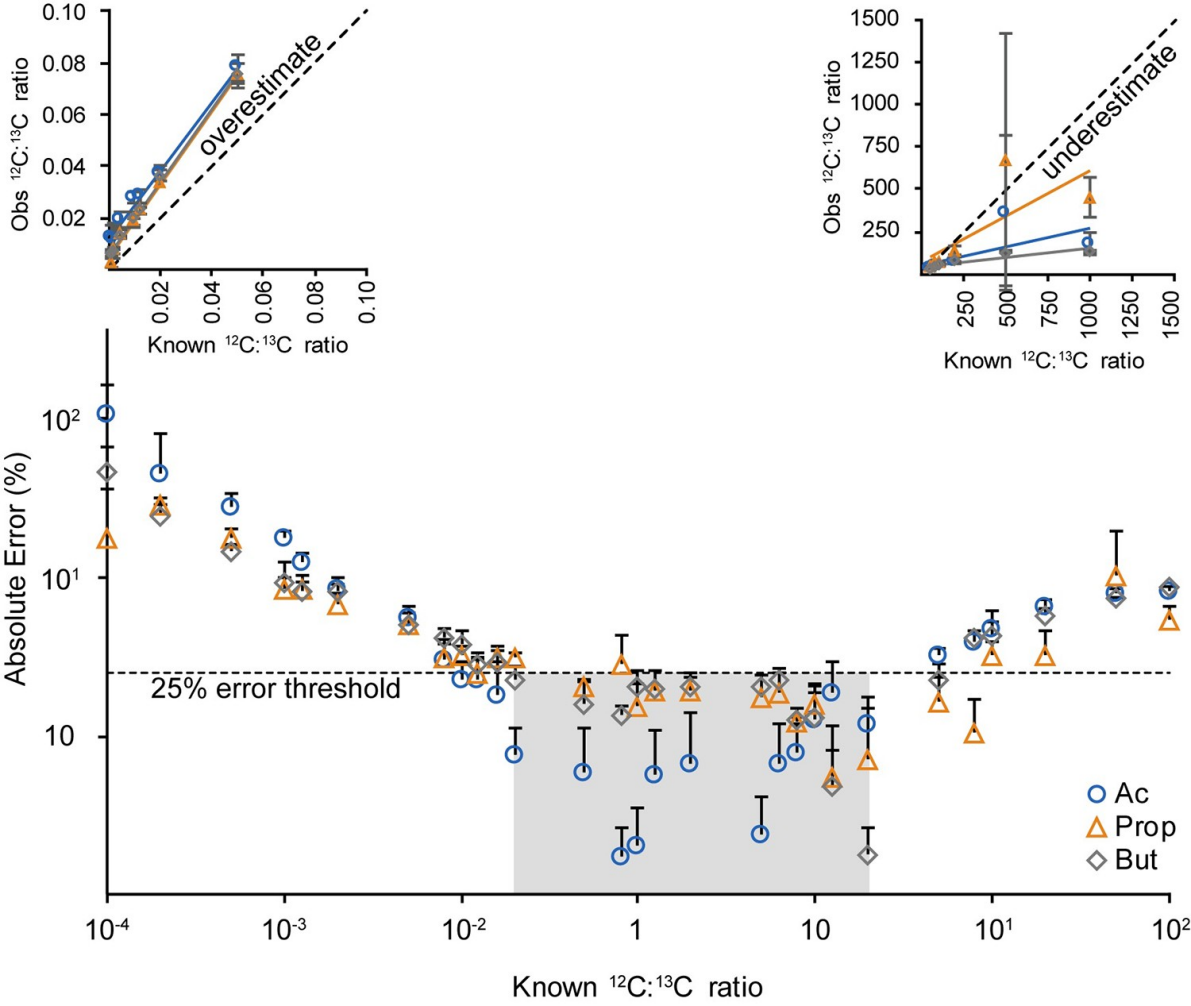
Relationship between ^12^C:^13^C isotope ratio for SCFAs and the observed absolute error. The dotted line denotes an arbitrary 25% error threshold. Colored square highlights the range of concentration ratios (0.5 < ^12^C:^13^C ratio < 20) where the absolute error is below 25%. (Insets show plots for the observed MS signal intensity ratio versus the known concentration ratio for (left) concentration ratios between 0.001 and 0.05 and (right) concentration ratios between 50 and 1000). Error bars indicate standard deviation, *n* = 3 technical replicates.

Although SQUAD can correct for most sources of experimental error, one important variable affecting these experiments is the ratio between ^12^C- and ^13^C-labeled analytes. To assess the impact these isotope ratios have on SCFA quantification, we prepared mixtures of standards with concentration ratios ranging from 0.001 to 1000 and quantified observed versus expected ratios for each compound to calculate error. We found that isotope ratios between 0.5 and 20 were necessary to maintain absolute error below 25% (Fig 3). Mixing ratios of ^12^C:^13^C below 0.5 overestimate isotope ratios, whereas ^12^C:^13^C mixing ratios above 20 underestimate the actual ratio (Fig 3, insets). These findings indicate that a two-stage workflow may be necessary for robust quantification of SCFAs in biological samples: the first stage used to establish rough concentration ranges for each endogenous analyte and the second with mixing isotope ratios between 0.5 and 20 for robust quantification.

### Quantification of microbial SCFA production in mouse caecum and *in vitro* cultures

**Fig 4:**
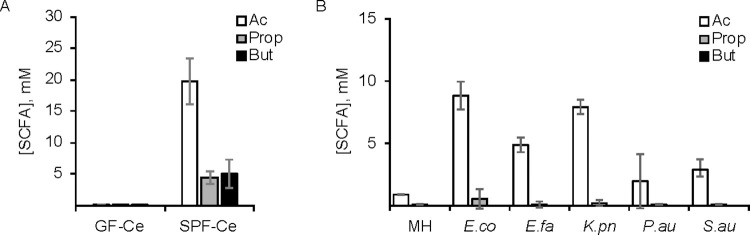
Microbial production of SCFAs in mice and *in vitro* cultures. SQUAD was used to analyze (A) caecal samples from germ free (GF, *n* = 5) and specific-pathogen-free (SPF, *n* = 4) mice, and (B) growth media of five species of microbes cultured for 4 hours in Mueller Hinton medium (*n* = 4 isolates per species). Error bars denote standard deviation. Abbreviations: MH, Mueller Hinton medium; *E*.*co*, *Escherichia coli*, *E*.*fa*, *Enterococcus faecalis*; *K*.*pn*, *Klebsiella pneumoniae*; *P*.*au*, *Pseudomonas aeruginosa; S*.*au*, *Staphylococcus aureus*.

One of the primary biological drivers for SCFA analyses is in the field of microbiome research, where microbial-derived SCFAs can have a significant impact on host biology. To test the biological applicability of SQUAD, levels of SCFAs in the caecal contents of germ free (GF) and specific pathogen free (SPF) mice were analyzed. Since gut microbes are the primary producers of SCFAs in mammals, we anticipated that GF mice would show minimal SCFA content relative to their SPF counterparts [24]. To test this, caecal contents were collected and SCFAs were extracted with a solvent spiked with ^13^C-internal standards. Caecal extracts were then derivatized, analyzed by LC-MS/MS, and endogenous SCFA levels were quantified. As predicted, the caecal extract of GF mice showed low levels of SCFAs. Acetate concentration was found to be 100 μM in the extract whereas propionate and butyrate had concentrations approaching the limit of detection. In contrast, the caecal contents of SPF mice contained all three SCFAs at over 200-fold greater levels than observed in GF mice (Fig 4A). In SPF mice, the relative abundance of these SCFAs followed a 4:1:1 ratio for acetate, propionate and butyrate, respectively. Both the higher abundance of SCFAs in SPF mice and the relative ratios of SCFAs we report here match the patterns reported elsewhere [25,26].

**Fig 5:**
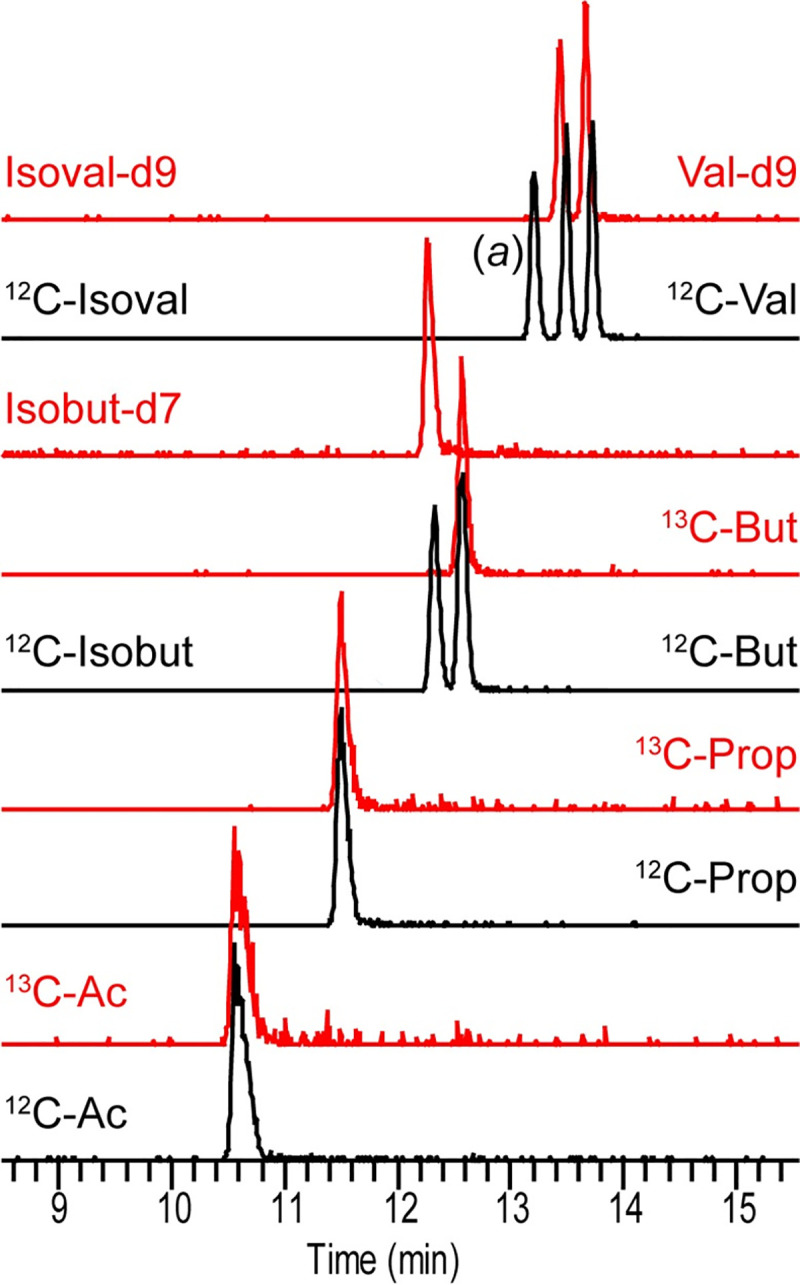
Extension of SQUAD to other SCFAs. Overlaid EICs corresponding to a ^12^C- (10 μM) and stable isotope labeled-(2 μM) derivatized SCFA standard mix solution detected in selected reaction monitoring mode. Red HPLC traces correspond to the ^13^C- or deuterated derivatized standards. Ac, Prop and But as previously described. (*a*) Indicates the HPLC trace for the 2-methylbutyrate standard (an isomer of valerate and isovalerate). Abbreviations: Isobut, isobutyrate; Val, valerate; Isoval, isovalerate.

One convenient aspect of the SQUAD workflow is that internal standards are added to biological samples prior to sample processing. This enables SQUAD to robustly quantify SCFAs from a wide diversity of biological source materials without needing to re-calibrate the assay for each individual application. To illustrate this point, we used SQUAD to analyze SCFAs produced by *in vitro* microbial cultures. Interestingly, although all five species cultured in this study secreted acetate, both propionate and butyrate levels were mostly unchanged compare to their level in the growth medium (110 μM and 8 μM, respectively) across the range of tested isolates (Fig 4B). These data highlight the flexible nature of SQUAD and the ability to adapt this method to diverse biological applications. To facilitate the use and adaption of this method to other applications, we have provided practical guideline for SQUAD analysis in the Supporting Information section (see S1 Appendix). Although SQUAD is presented here as a targeted assay for gut microbiome research, and consequently has relatively narrow range of example compounds, the method could be expanded to a wider transect of metabolites provided that isotope-labeled standards are available (e.g., isobutyrate, valerate and isovalerate; Fig 5).

## Conclusion

Herein, we introduce SQUAD, an isotope-based strategy for absolute quantification of SCFAs in complex biological samples. The primary advantage of SQUAD is that it is not sensitive to incomplete derivatization and other chemistry-related variables that affect existing SCFA quantification methods. We demonstrate that SCFAs can be quantified with less than 25% error provided that ^12^C:^13^C mixing ratios are maintained between 0.5 and 20. Moreover, we demonstrate that the SQUAD approach is readily amenable to a diverse range of biological applications. Although the SQUAD approach we present here is primarily focused on SCFA quantification, the underlying aniline derivatization approach is applicable to a wide range of biomolecules containing carboxylic acids [18]. In summary, SQUAD enables robust LC-MS/MS quantification of the primary molecules of interest to the microbiome community with minimal sources of error.

## Supporting information

S1 Fig Calibration curves, LLOD and LLOQ determination for derivatized SCFA standards. (PDF)

S2 Fig Effect of the EDC molar equivalent on observed ^12^C:^13^C ratio. (PDF)

S3 Fig Effect of the derivatization reaction time on observed ^12^C:^13^C ratio. (PDF)

S4 Fig Effect of the extraction solvent composition on observed ^12^C:^13^C ratio. (PDF)

S1 Table Concentrations of ^12^C- and ^13^C-standard solutions used to make up indicated ^12^C:^13^C concentration ratios. (PDF)

S2 Table Caecal samples and extraction details. (PDF)

S3 Table Regression line equations and r^2^ corresponding to plots in Fig 2. (PDF)

S4 Table Calculation of the number of moles of EDC relative to the total number of moles of SCFAs. (PDF)

S1 Appendix Practical guidelines for quantification of SCFAs. (PDF)
