# Supplementary material for: Method for absolute quantification of short chain fatty acids via reverse phase chromatography mass spectrometry
Source: PLoS One. 2022 Apr 20;17(4):e0267093. doi: 10.1371/journal.pone.0267093 (PMC9020710; doi:10.1371/journal.pone.0267093)
Supplement: S1 Fig — (PDF) [file pone.0267093.s001.pdf]

A

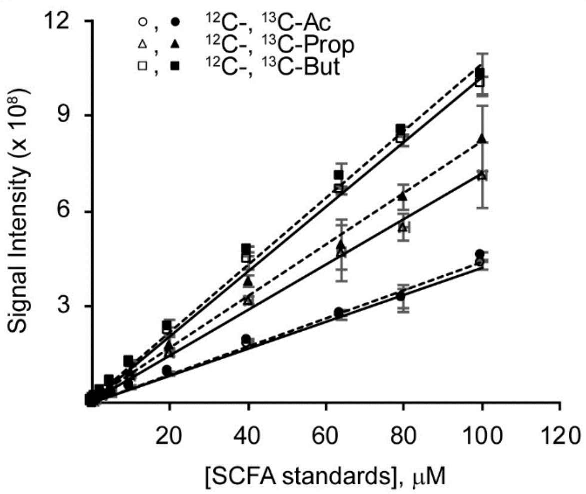

B

Standards	Regression line equation	r^2
^{12}C-Ac	y = 4.24E+06x + 2.29E+06	0.998
^{13}C-Ac	y = 4.38E+06x + 3.04E+06	0.995
^{12}C-Prop	y = 7.12E+06x + 5.71E+06	0.997
^{13}C-Prop	y = 8.16E+06x + 6.33E+06	0.996
^{12}C-But	y = 1.01E+07x + 8.23E+06	0.998
^{13}C-But	y = 1.06E+07x + 1.04E+07	0.997

C

Analyte concentration (μM)	0.02	0.04	0.08	0.16	0.31
Acetate	1.6	**3.3**	4.0	6.4	**10.2**
Propionate	2.3	**3.6**	4.3	**10.4**	21.9
Butyrate	2.6	**3.4**	7.0	**10.3**	14.8

S1 Fig. Calibration curves, LLOD and LLOQ determination for derivatized SCFA standards. (A) Calibration curves for the 3 isotopologue pairs of SCFA standards generated from the derivatization of an equimolar standard solution. Concentrations ranged from 20 nM to 100 μM. Dotted lines represent ^{13}C-standards. Error bars indicate standard deviation, (*n* = 3 technical replicates). MS signal intensities are plotted in arbitrary units (a. u.). (B) Regression line equations and r^2 corresponding to plots in (A). (C) Signal-to-noise ratio (S/N) from chromatographic peaks for 3 analytes (acetate, propionate and butyrate derivatized with aniline) at five different concentrations (0.02, 0.04, 0.08, 0.16 and 0.31 μM, in H_2O/ACN (50:50, v/v)). Bold figures indicate the signal-to noise ratios used for the determination of the LLOD and LLOQ for the analytical method (≥ 3 and ≥ 10, respectively).
