# Supplementary material for: Method for absolute quantification of short chain fatty acids via reverse phase chromatography mass spectrometry
Source: PLoS One. 2022 Apr 20;17(4):e0267093. doi: 10.1371/journal.pone.0267093 (PMC9020710; doi:10.1371/journal.pone.0267093)
Supplement: S2 Fig — (PDF) [file pone.0267093.s002.pdf]

A

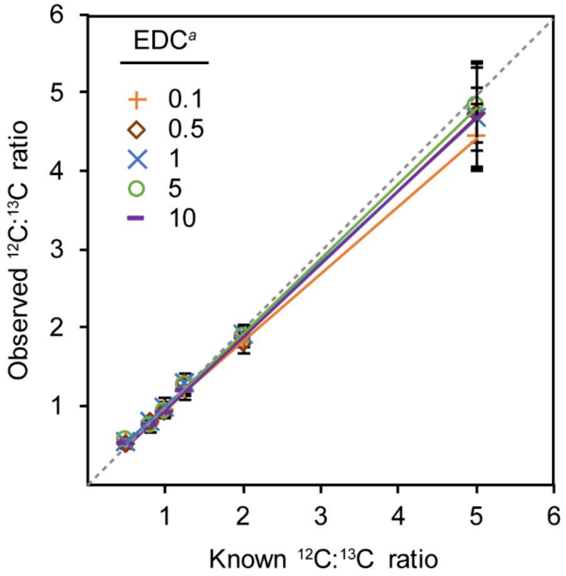

B

EDC[a]	Regression line equation	r^2
0.1	y = 0.8643x + 0.1050	0.999
0.5	y = 0.9364x + 0.0238	0.998
1	y = 0.9236x + 0.0696	0.999
5	y = 0.9592x + 0.0124	0.999
10	y = 0.9422x - 0.0102	0.999

[a] expressed as molar equivalent compared to the total number of moles of SCFAs.

S2 Fig. Effect of the EDC molar equivalent on observed ^{12}C:^{13}C ratio. (A) Five different molar equivalents (0.1, 0.5, 1, 5 and 10) of EDC were each tested on six different ^{12}C/^{13}C SCFA standard mixed solutions with a ^{12}C- and ^{13}C- standards concentration ratio of 0.5, 0.8, 1, 1.25, 2 and 5. Dashed line represents a slope = 1. Error bars represent standard deviation, averaged data for the three SCFAs. (B) Regression line equations and r^2 corresponding to plots in (A).
