# Supplementary material for: Method for absolute quantification of short chain fatty acids via reverse phase chromatography mass spectrometry
Source: PLoS One. 2022 Apr 20;17(4):e0267093. doi: 10.1371/journal.pone.0267093 (PMC9020710; doi:10.1371/journal.pone.0267093)
Supplement: S3 Fig — (PDF) [file pone.0267093.s003.pdf]

A

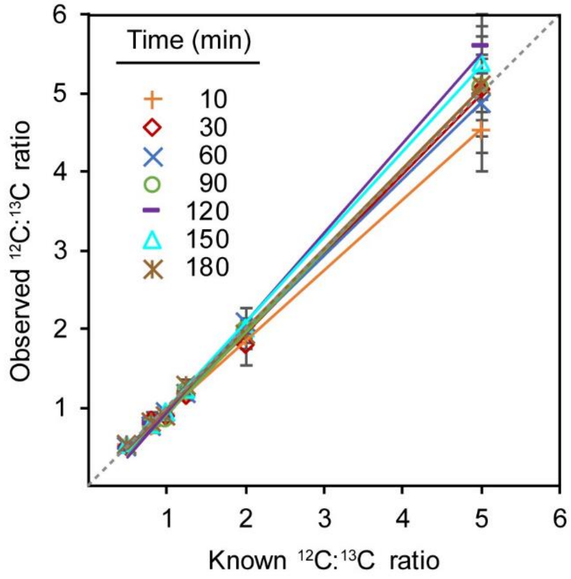

B

Reaction time (min)	Regression line equation	r^2
10	y = 0.8954x + 0.0643	0.999
30	y = 1.0150x - 0.0796	0.997
60	y = 0.9759x + 0.0026	0.998
90	y = 1.0368x - 0.1122	0.999
120	y = 1.1463x - 0.2131	0.997
150	y = 1.0907x - 0.1075	0.999
180	y = 1.0218x - 0.0458	0.999

C

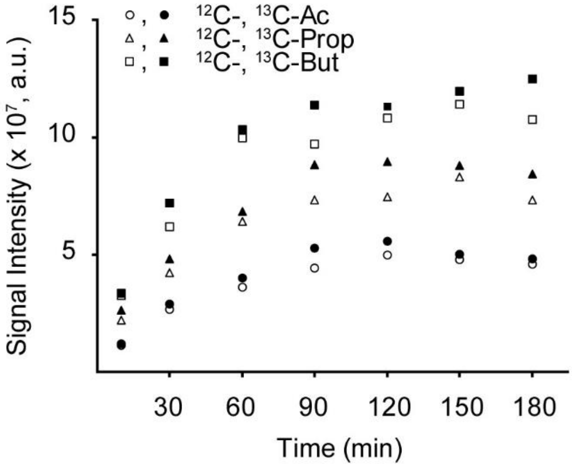

S3 Fig. Effect of the derivatization reaction time on observed ^{12}C:^{13}C ratio. (A) Derivatization was carried out on six different $^{12}C/^{13}C$ SCFA standard mixed solutions with a ^{12}C:^{13}C concentration ratio of 0.5, 0.8, 1, 1.25, 2 and 5. Aliquots were sampled over time. Dashed line represents a slope = 1. Error bars represent standard deviation, averaged data for the three SCFAs. (B) Regression line equations and r^2 corresponding to plots in (A). (C) MS Signal intensity is plotted over time for the 3 standard isotopologue pairs present in an equimolar ratio in the sample; expressed in arbitrary units (a.u.).
