# Supplementary material for: Method for absolute quantification of short chain fatty acids via reverse phase chromatography mass spectrometry
Source: PLoS One. 2022 Apr 20;17(4):e0267093. doi: 10.1371/journal.pone.0267093 (PMC9020710; doi:10.1371/journal.pone.0267093)
Supplement: S4 Fig — (PDF) [file pone.0267093.s004.pdf]

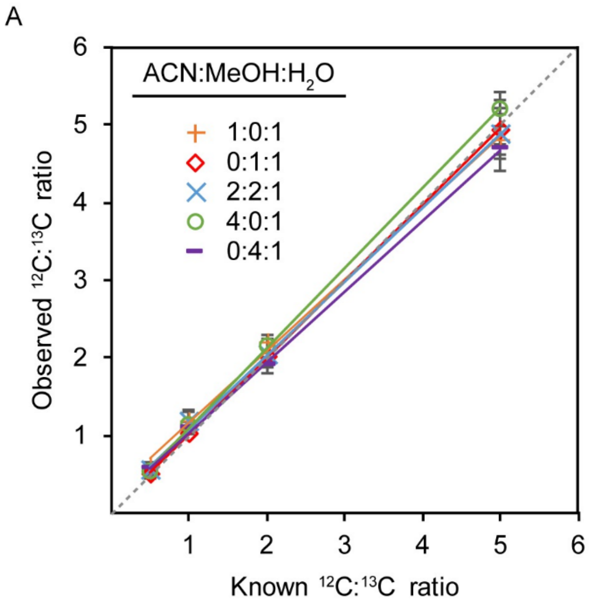

B

ACN:MeOH:H_2O (v:v:v)	Regression line equation	r^2
1:0:1	y = 0.9208x + 0.2367	0.998
0:1:1	y = 0.9830x + 0.0266	1
2:2:1	y = 0.9477x + 0.1387	0.999
4:0:1	y = 1.0292x + 0.0598	0.999
0:4:1	y = 0.9089x + 0.1287	0.999

S4 Fig. Effect of the extraction solvent composition on observed ^{12}C:^{13}C ratio. (A) Four different ^{12}C/^{13}C SCFA standard mixed solutions, containing ^{12}C-acetate, ^{12}C -propionate and their corresponding ^{13}C-counterparts were prepared, with a ^{12}C:^{13}C concentration ratio of 0.5, 1, 2 and 5, in five different reaction/organic solvents, namely H_2O/ACN (50:50, v/v), H_2O/ACN (20:80, v/v), H_2O/MeOH (50:50, v/v), H_2O/MeOH (20:80, v/v) and H_2O/MeOH/ACN (20:40:40, v/v). Dashed line represents a slope = 1. Error bars represent standard deviation, averaged data for the two tested standards (acetate and propionate). (B) Regression line equations and r^2 corresponding to plots in (A).
