# Supplementary material for: Method for absolute quantification of short chain fatty acids via reverse phase chromatography mass spectrometry
Source: PLoS One. 2022 Apr 20;17(4):e0267093. doi: 10.1371/journal.pone.0267093 (PMC9020710; doi:10.1371/journal.pone.0267093)
Supplement: S1 Table — (PDF) [file pone.0267093.s005.pdf]

S1 Table.Concentrations of ^{12}C- and ^{13}C-standard solutions used to make up indicated ^{12}C:^{13}C concentration ratios.

[^{12}C-SCFA]: [^{13}C-SCFA]	[^{12}C-SCFA], μM	[^{13}C-SCFA], μM
0.001	1	1000
0.002	2	1000
0.005	5	1000
0.01	10	1000
0.0125	12.5	1000
0.02	20	1000
0.05	50	1000
0.08	80	1000
0.1	100	1000
0.125	125	1000
0.16	160	1000
0.2	200	1000
0.5	500	1000
0.8	800	1000
1	1000	1000
1.25	1000	800
2	1000	500
5	1000	200
6.25	1000	160
8	1000	125
10	1000	100
12.5	1000	80
20	1000	50
50	1000	20
80	1000	12.5
100	1000	10
200	1000	5
500	1000	2
1000	1000	1
