# Supplementary material for: Method for absolute quantification of short chain fatty acids via reverse phase chromatography mass spectrometry
Source: PLoS One. 2022 Apr 20;17(4):e0267093. doi: 10.1371/journal.pone.0267093 (PMC9020710; doi:10.1371/journal.pone.0267093)
Supplement: S2 Table — (PDF) [file pone.0267093.s006.pdf]

**S2 Table. Caecal samples and extraction details.**

| Location | Flora | wet weight (mg) | Extraction solvent (µL) |
|---|---|---|---|
| Caecum | SPF | 88.7 | 177.4[a] |
| Caecum | SPF | 59.9 | 119.8 [a] |
| Caecum | SPF | 86.1 | 172.2 [a] |
| Caecum | SPF | 84.3 | 168.6 [a] |
| Caecum | GF | 137.4 | 274.8 [b] |
| Caecum | GF | 210.0 | 420.0 [b] |
| Caecum | GF | 172.7 | 345.4 [b] |
| Caecum | GF | 106.4 | 212.8 [b] |
| Caecum | GF | 141.5 | 283.0 [b] |

[a] IS spiked-in concentrations: 10 mM $^{13}$C-Acetate / 1 mM $^{13}$C-Propionate / 2.5 mM $^{13}$C-Butyrate.

[b] IS spiked-in concentrations: 100 µM $^{13}$C-Acetate / 50 µM $^{13}$C-Propionate / 50 µM $^{13}$C-Butyrate.
