# Supplementary material for: Method for absolute quantification of short chain fatty acids via reverse phase chromatography mass spectrometry
Source: PLoS One. 2022 Apr 20;17(4):e0267093. doi: 10.1371/journal.pone.0267093 (PMC9020710; doi:10.1371/journal.pone.0267093)
Supplement: S3 Table — (PDF) [file pone.0267093.s007.pdf]

S3 Table. Regression line equations and r^2 corresponding to plots in Figure 2.

SCFA	Regression line equation	r^2
Acetate	y = 0.9119x + 0.2089	0.996
Propionate	y = 1.0628x + 0.3525	0.998
Butyrate	y = 1.0078x + 0.5665	0.991
