# Supplementary material for: Method for absolute quantification of short chain fatty acids via reverse phase chromatography mass spectrometry
Source: PLoS One. 2022 Apr 20;17(4):e0267093. doi: 10.1371/journal.pone.0267093 (PMC9020710; doi:10.1371/journal.pone.0267093)
Supplement: S4 Table — (PDF) [file pone.0267093.s008.pdf]

S4 Table. Calculation of the number of moles of EDC relative to the total number of moles of SCFAs.

	[SCFAs], M		n (mole)		Volume (µL)
[^{12}C-SCFA]:[^{13}C-SCFA]	^{12}C-SCFAs	^{13}C-SCFAs	SCFAs (total)	EDC	EDC
**0.50**	5.E-04	1.E-03	**4.5E-07**	4.5E-06	**3.75**
**0.80**	8.E-04	1.E-03	**5.4E-07**	5.4E-06	**4.50**
**1.00**	1.E-03	1.E-03	**6.0E-07**	6.0E-06	**5.00**
**1.25**	1.E-03	8.E-04	**5.4E-07**	5.4E-06	**4.50**
**2.00**	1.E-03	5.E-04	**4.5E-07**	4.5E-06	**3.75**
**5.00**	1.E-03	2.E-04	**3.6E-07**	3.6E-06	**3.00**

Note: Calculation are shown for six mix standard solutions with an isotope ratio ranging from 0.5 to 5, and based on 100 µl of SCFA solution and 10 molar equivalent of 1.2 M EDC.
Numbers in bold are mentioned in the study of the "Effect of the EDC molar equivalent on observed ^{12}C:^{13}C ratio" described in the "Materials and methods" section.
