# Supplementary material for: Method for absolute quantification of short chain fatty acids via reverse phase chromatography mass spectrometry
Source: PLoS One. 2022 Apr 20;17(4):e0267093. doi: 10.1371/journal.pone.0267093 (PMC9020710; doi:10.1371/journal.pone.0267093)
Supplement: S1 Appendix — (PDF) [file pone.0267093.s009.pdf]

S1 Appendix. Practical guidelines for quantification of SCFAs.

^{13}-SCFA standards spiked-extraction solvent

- Prepared from freshly made up individual stock solution of 20 mM ^{13}C-acetate, 10 mM ^{13}Cpropionate, 10 mM ^{13}C-butyrate (1.5 mL each). Volume needed for each standard is between 1-2 µL, measured with a P2 pipette for better accuracy. Individual stock solutions are further diluted as needed, and mixed to give working solutions which are kept on ice until used.

Positive controls:

- Prepared each time from freshly made up individual stock solution of 20 mM ^{13}C-acetate, 10 mM ^{13}C-propionate, 10 mM ^{13}C-butyrate (1.5 mL each).
- Required additional ^{12}C-SCFA mixed standard stock solution (100 µL at 200 mM final concentration for each standard)
- Using the four above-mentioned stock solutions, prepare 4 samples (400 µL) with ^{12}C:^{13}C concentration of 1 mM:2 mM, 1 mM:1mM, 1 mM:400 µM and 1 mM:200 µM (corresponding concentration ratio of 0.5, 1, 2.5 and 5, respectively).
- Dilute samples to 1:100 with 50% H_2O/MeOH (v/v) before LC-MSMS analysis.

Back calculation of spiked-in ^{13}C-SCFA standard concentrations:

- ^{13}C-SCFA standards spiked-extraction solution is spiked with the ^{12}C-SCFA mixed standard stock solution (200 mM final concentration for each standard) diluted to a practical concentration to give a final ^{13}C:^{12}C concentration ratio, for each isotopologue pair, within a 0.5-20 range.
- Dilute sample(s) as needed with 50% H_2O/MeOH (v/v) before LC-MSMS analysis.

Biological considerations:

SCFAs levels can span many orders of magnitude between biological groups (e.g SPF versus GF mice). Pilot projects should include each condition to calibrate isotope spiking ratios and sample dilution prior to LC-MS/MS analysis
